# Supplementary material for: An ensemble code in medial prefrontal cortex links prior events to outcomes during learning
Source: Nat Commun. 2018 Jun 7;9:2204. doi: 10.1038/s41467-018-04638-2 (PMC5992197; doi:10.1038/s41467-018-04638-2)
Supplement: Supplementary file 1 — Supplementary Information [file 41467_2018_4638_MOESM1_ESM.pdf]

Supplementary Information

Title: "An ensemble code in medial prefrontal cortex links prior events to outcomes during learning"
Authors: Maggi et al

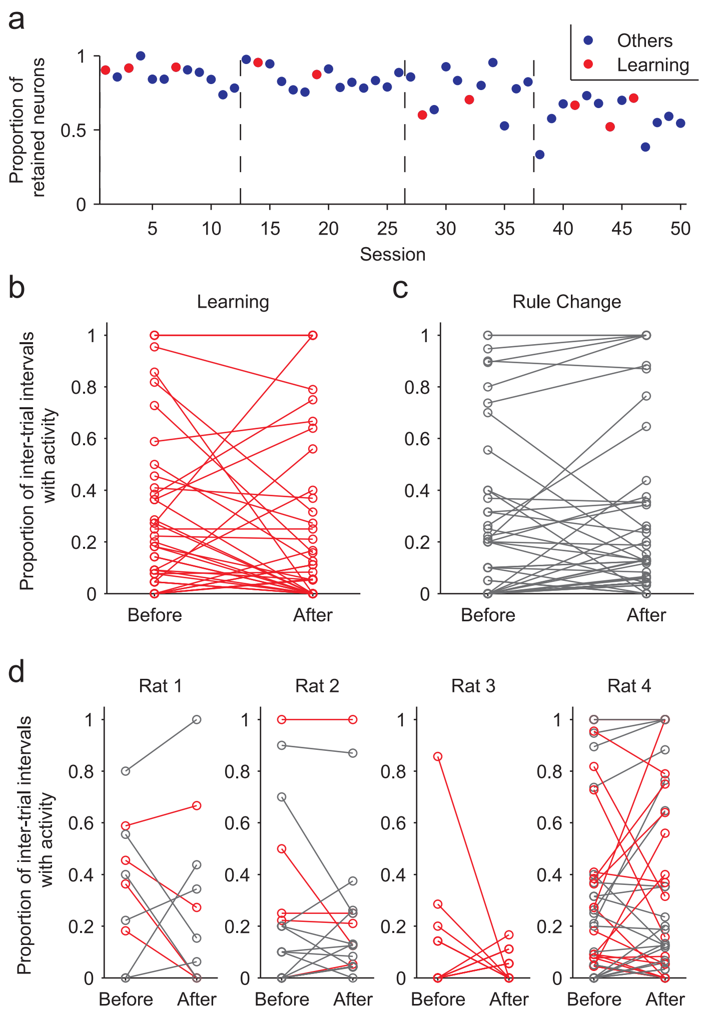

Supplementary Figure 1. Population sizes did not differ between session types.
(**a**) The proportion of neurons retained in the core population for each session. Retained neurons were those that fired in every inter-trial interval. The black vertical dashed lines separate the sessions for each of the four rats.
(**b**) For learning sessions, the proportion of inter-trial intervals in which each non-core neuron (circle) was active before and after the learning trial. We see no systematic recruitment (an increase in proportion) or suppression (decrease in proportion) of the non-core neurons by learning.
(**c**) As panel **b**, comparing before and after the rule-change trial in rule change sessions. Again we see no systematic recruitment or suppression of the non-core neurons by a rule-change.
(**d**) Breakdown of changes in panels **b** and **c** for each rat, showing no individual differences in the recruitment of non-core neurons.

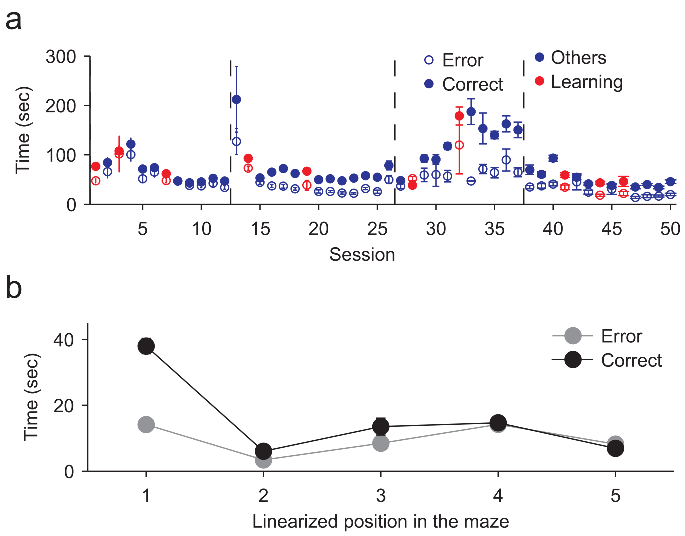

Supplementary Figure 2. Statistics of time periods during the inter-trial intervals for the 50 retained sessions.

(**a**) Durations of the inter-trial intervals within each session, given as the mean ± SEM duration in seconds, separated into post-correct (filled symbols) and post-error (open symbols) inter-trial intervals. Red symbols are the learning sessions. Vertical dashed lines separate sessions by rat.

(**b**) Time spent along the maze during the inter-trial intervals, given as the mean ± SEM seconds spent across all the animals and all the sessions for post-correct (black) and post-error (grey) inter-trial intervals. The maze has been linearised and divided in 5 equal sized sections, with position 1 being the reward location, position 3 the choice point of the Y-maze, and position 5 the end of the start arm – see Figure 5 (main text) for a schematic.

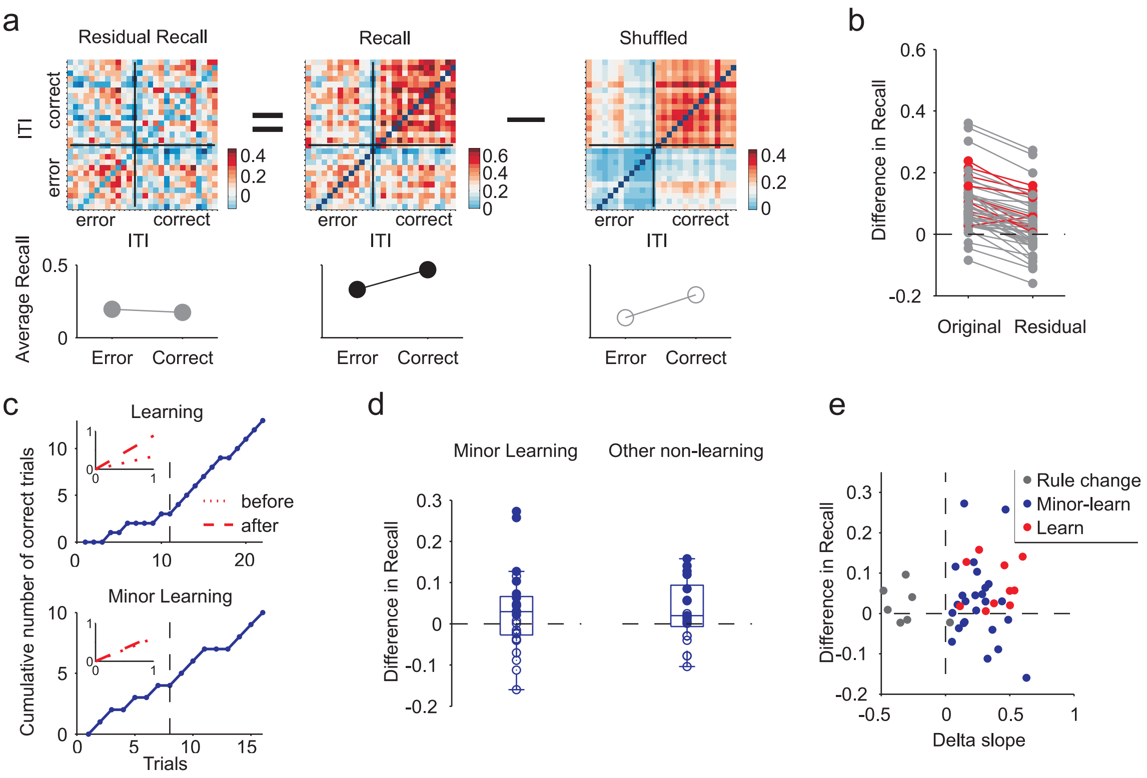

Supplementary Figure 3. Recall of neural ensembles is learning-specific.
(**a**) Example correction of the recall matrix to remove the confounding effect of different durations of the post-correct and post-error inter-trial intervals. The residual recall matrix was obtained as the difference between the recall matrix and the mean matrix obtained from the shuffled inter-spike intervals (upper panels). In this example session, the difference between average recall values for post-error and post-correct intervals was about the same for the data and shuffled data (bottom panels). Consequently, for this session we could account for the higher recall following reinforcement entirely by the differing durations of the post-error and post-correct intervals.
(**b**) Comparison of the difference between average correct recall and error recall before ('Original') and after ('Residual') correction by the shuffled control data. Red symbols are the learning sessions.
(**c**) To check whether the recall effect was specific to sessions showing abrupt learning (top panel; Figure 2, main text), we identified a subset of the other sessions with potential incremental or "minor" learning. These minor-learning sessions were any in which the curve of cumulative rewards contained a detectable upward inflection, as shown by the existence of any trial with a greater slope in a regression line after that trial than before it (insets, red lines). The vertical black dashed line is the identified learning trial.
(**d**) The difference between average correct recall and error recall for the minor-learning and remaining other sessions. No systematic recall effect was observed for the minor-learning sessions, suggesting the recall effect was specific to abrupt transitions in behaviour.
(**e**) Relationship between behavioural change and the strength of recall. The difference between average correct recall and error recall is plotted as a function of the difference between the slopes of the fitted lines before and after the learning trial (Delta slope). Sessions: learning (red), rule change (grey), and minor-learning (blue). Delta slope for each rule change session was computed with respect to the rule change trial.

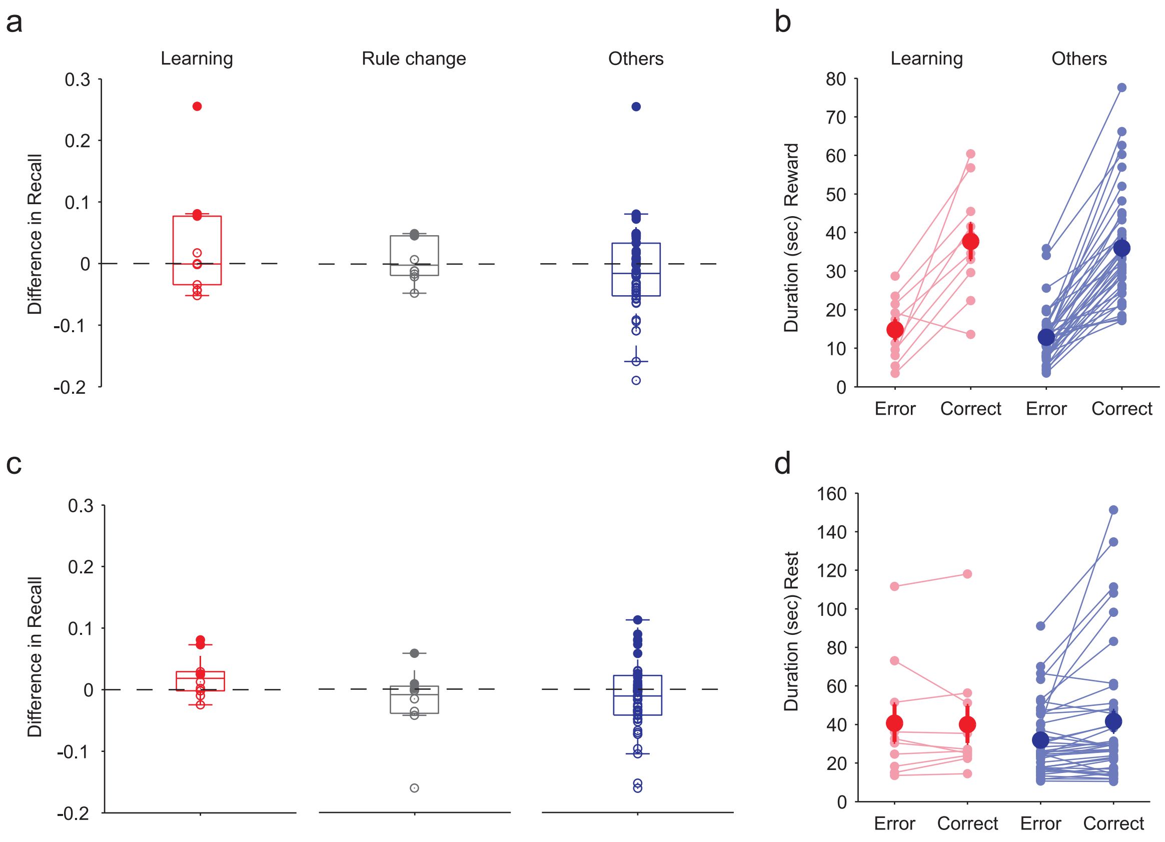

Supplementary Figure 4. Neural ensemble recall is not specific to reward consumption.
(**a**) The difference in average recall between post-error and post-correct intervals when using neural activity only at the reward location (position 1 in Figure 5a). No systematic post-reinforcement recall occurs at the reward location, for any session type.
(**b**) Duration of time spent by the animals in the reward location (position 1 in Figure 5a) for post-error and post-correct inter-trial intervals. Learning sessions (red) and all other sessions (blue) have similar average durations of time in the reward location for post-correct intervals and for post-error intervals (note that all others here include "rule change" and "others" of panel **a**). Each pair of dots is a session. The bold symbols are mean ± SEM of the entire distribution.
(**c**) As panel **a**, but using neural activity only during the self-paced return (positions 2-5 in Figure 5a) to the start position. The learning sessions show a weakly-positive reinforcement-specific recall.
(**d**) As panel **b**, for the duration of the self-paced return across positions 2-5 in the maze.

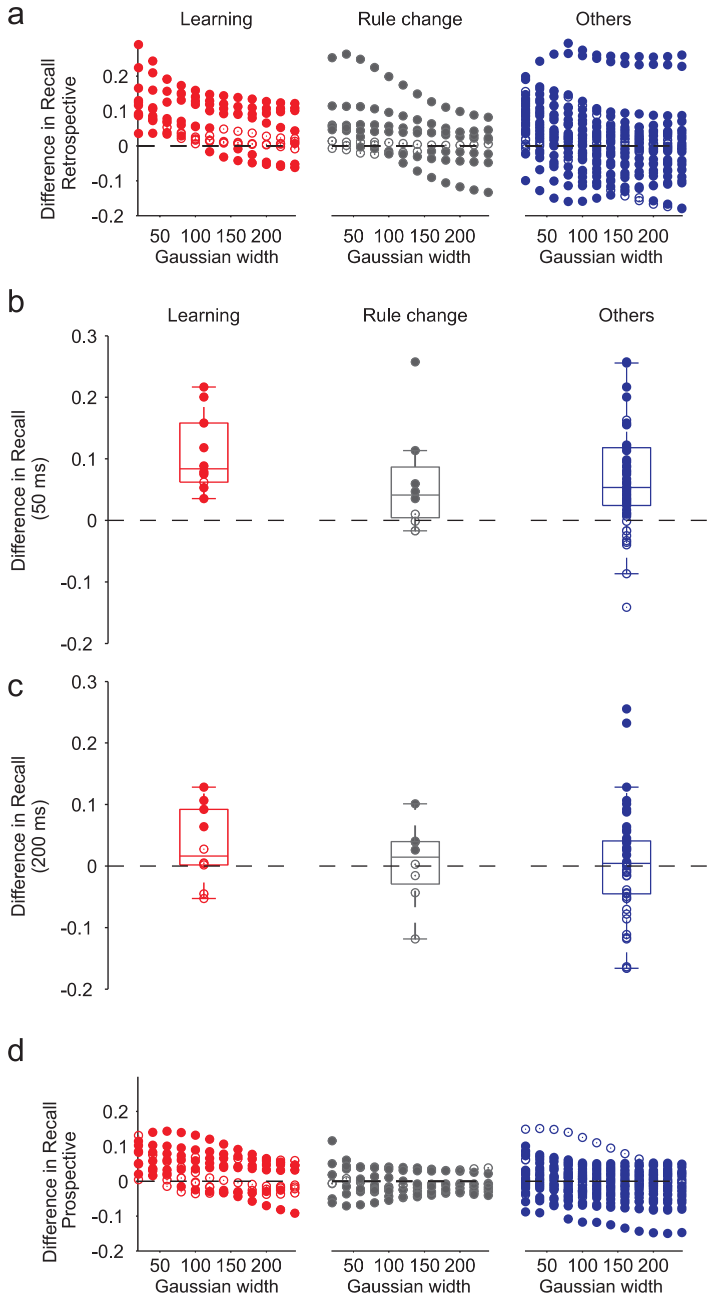

Supplementary Figure 5. Time-scale dependence of recall.
(**a**) Dependence of the recall of ensemble activity on the temporal precision of spike-train correlation. Here we plot the distribution of the mean difference in recall between post-correct and post-error interbals as a function of the Gaussian width used to convolve the spike-trains. A difference in recall greater than zero indicates the interval similarity matrices were more similar for correct than for error intervals. Each symbol is one session. Filled circles indicate a difference at $P < 0.05$ between the distributions of recall values in the error and correct intervals (Kolmogorov-Smirnov test).
(**b**) Comparison of the mean difference in recall across learning, rule change, and other sessions after spike-train convolution with a Gaussian 50 ms wide.
(**c**) As for panel **b**, but for a Gaussian 200 ms wide.
(**d**) Dependence of the prospective recall of ensemble activity on the temporal precision of spike-train correlation. As for panel a, but here we plot the difference in prospective recall, between intervals before correct and before error trials. We only see a systematic recall before reinforcement at the smaller tested Gaussian widths (≤ 40 ms), which is likely a reflection of the stronger retrospective recall effect at these widths.

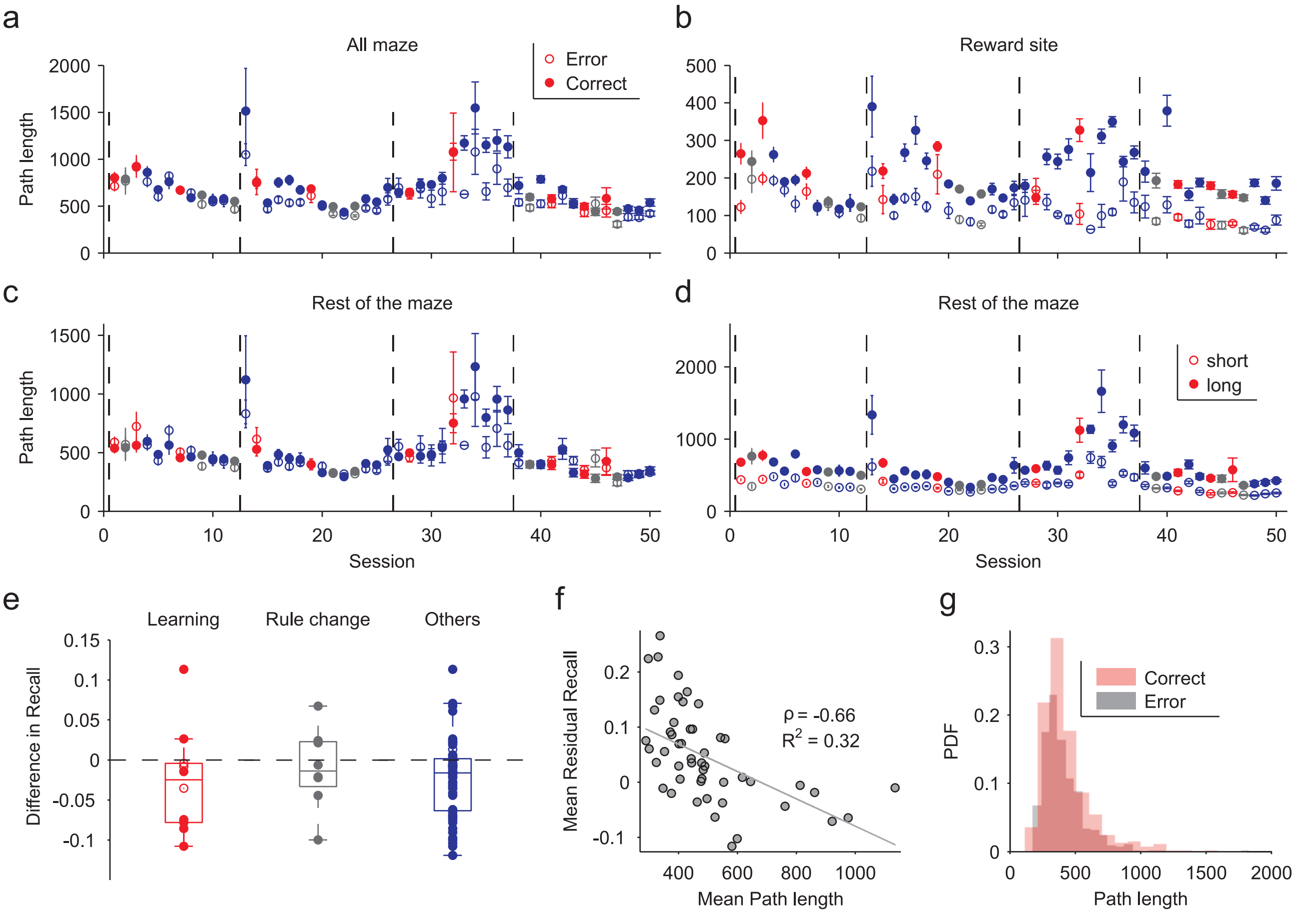

Supplementary Figure 6. Path length effects on the recall of ensemble activity patterns.
(**a**) Total path lengths of the trip from the reward location to the start point during inter-trial intervals, for error or correct trials. Plotted are mean ± SEM per session. Session types are colour-coded: learning (red); rule-change (grey); other sessions (blue). Vertical lines separate sessions for each rat.
(**b**) As for panel **a**, for path lengths only within the reward location (position 1 on the maze). Path lengths during reward consumption after correct trials (solid symbols) were typically longer, as expected.
(**c**) As for panel **b**, for path lengths after leaving the reward location on the return trip. There was no systematic difference between post-error and post-correct path lengths in the rest of the maze.
(**d**) Division of path lengths across the rest of the maze into two groups (short/long) according to the median path length in that session. Plotted are the mean ± SEM within each group. Analyses in panels e-g all use these rest-of-maze groups.
(**e**) Difference in residual recall between short and long path-length groups of inter-trial intervals, by session type. As per the outcome-dependent recall analysis (Figure 2, main text), we computed the mean recall between inter-trial intervals of the same group (short or long path-length), and plot here the difference between those means (short - long), one symbol per session. We see a small bias towards higher recall for short path lengths across all session types, suggesting a weak but persistent effect of path length.
(**f**) Mean recall is negatively correlated with mean path length. For each group of inter-trial intervals (whether "short" or "long"), we plot the mean recall within the group against its mean path length (from panel d). ρ: Spearman's rank correlation; R^2: robust linear regression.
(**g**) Rest-of-maze path length distributions over inter-trial intervals of the learning sessions. There is no difference in path length between inter-trial intervals following errors or correct choices; thus the observed difference in reinforcement-selective recall during learning sessions is unrelated to the weak path length effect common to all sessions.

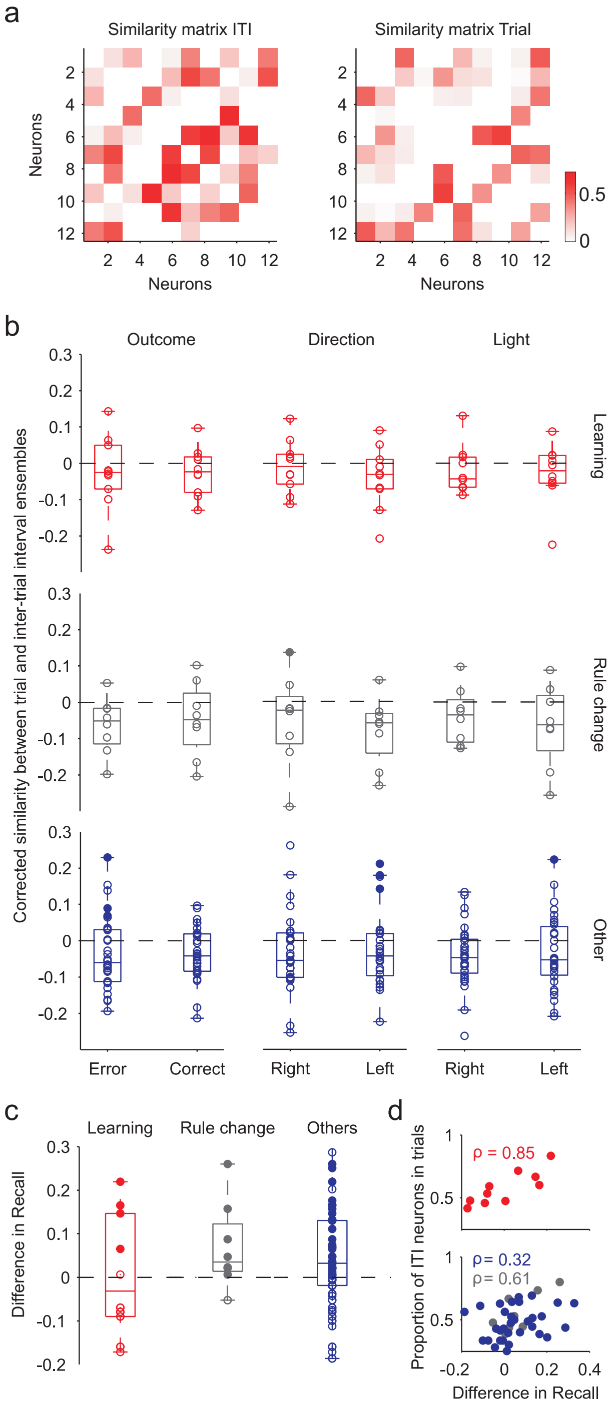

Supplementary Figure 7. Ensemble activity recalled within the inter-trial interval was not the preceding trial's ensemble activity.

(**a**) Example neural activity similarity matrices for an inter-trial interval (ITI) and for its preceding trial. We used here the core population of neurons active in every trial of a session. For each such pair of matrices we computed the similarity between them. To predict the similarity expected between independently firing ensembles, we also computed the similarity between the trial's matrix and each of a set of matrices from shuffled inter-trial intervals.

(**b**) Relative similarity between ensemble activity patterns of inter-trial intervals and preceding trials. In each scatter, a symbol is the mean difference between the data's similarity and the predicted similarity of independent ensembles: a difference greater than zero indicates the trial and inter-trial interval ensemble activity patterns are closer than predicted by independent patterns of firing. Distributions are centred around zero, indicating that the ensemble activity in the trial and the following inter-trial are not related. Solid symbol: session with $P < 0.05$ (signtest) for a difference greater than zero between data and shuffled similarities.

(**c**) Difference in recall between post-correct and post-error inter-trial intervals, computed using only the neurons retained for the trials' core population in each session.

(**d**) Relationship between the difference in recall (panel c) and the proportion of neurons in common between the core populations for the inter-trial intervals and for the trials. Recall was correlated with the size of the shared core population in learning sessions (top), and in the rule-change and other sessions (bottom), albeit more weakly. ρ: Spearmans rank correlation.

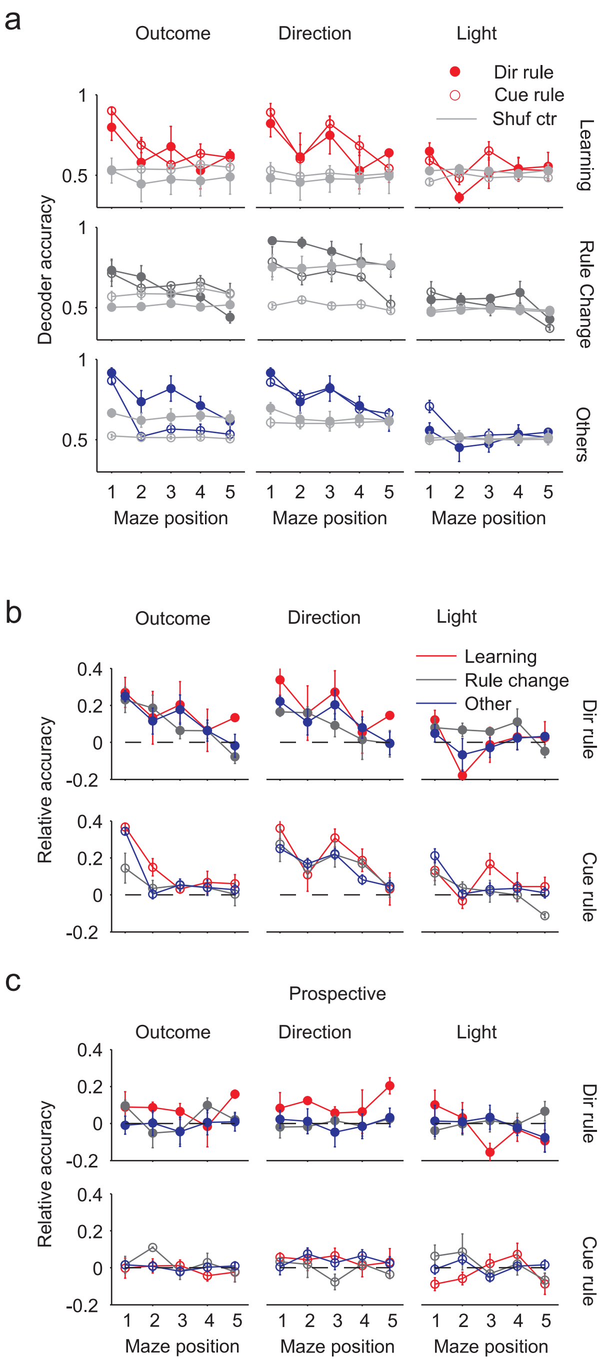

Supplementary Figure 8. Further decoding analysis.
(**a**) Decoding can be near perfect. Here we replot the breakdown of the decoding results in Figure 5**d** as the absolute accuracy of the decoders (where 1 is maximum, indicating correct prediction of every held-out inter-trial interval). Each data point is the mean ± SEM accuracy at that maze position The control results on shuffled inter-trial interval labels are shown as grey lines. The "other" sessions were plotted in Figure 5**c**.
(**b**) Comparison of above-chance decoding performance between the same rule types. Each data point is the mean ± SEM accuracy in excess of chance (dashed line) over the indicated combination of session type and rule type.
(**c**) Comparison of prospective decoding performance between the same rule types, confirming the absence of the prospective encoding of task-relevant information. Similar to panel **b**, here we plot the mean ± SEM above-chance accuracy of decoding prospective outcome, direction, or cue position, separately for sessions with direction or light rules. As in panel **b**, decoding accuracy is normalised by the corresponding shuffled control decoding performance (where 0 is identical to shuffled controls).

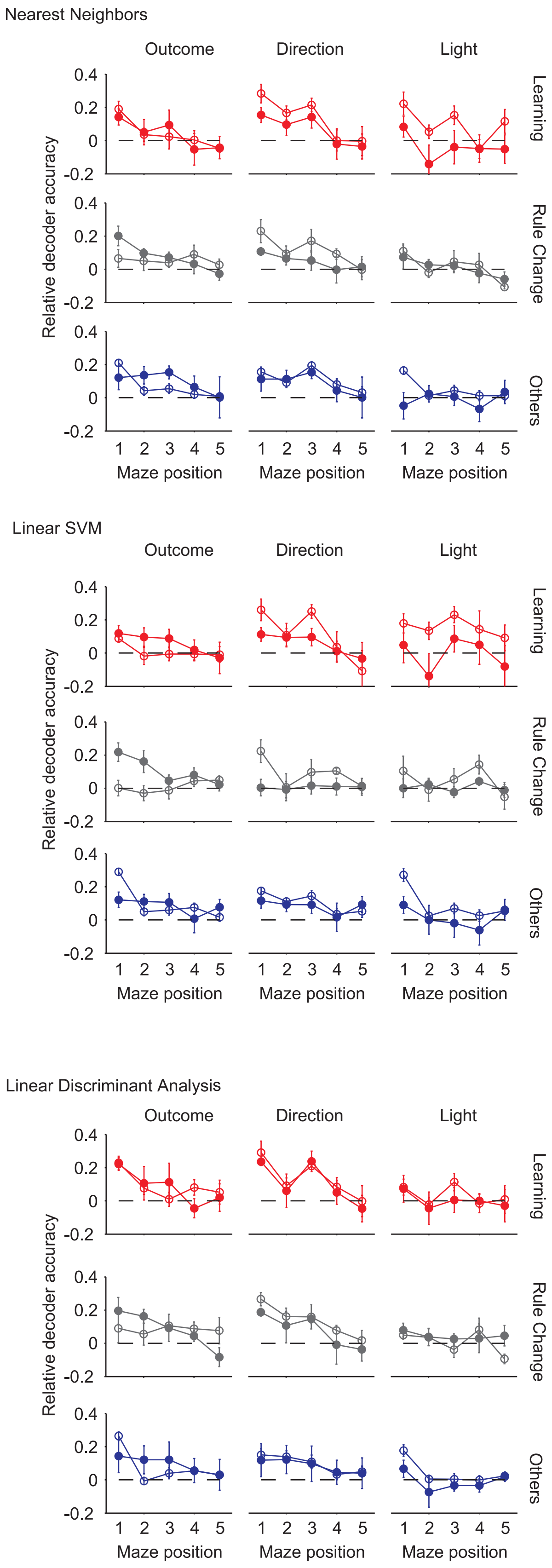

Supplementary Figure 9. Robustness of the retrospective encoding of task-relevant information. Using the same layout as Figure 5d, here we summarise the decoding performance of three further classifiers we tested on the data to check the robustness of the decoding results. Top: Nearest Neighbors; middle: linear Support Vector Machine; bottom: Linear Discriminant Analysis. Symbols plot mean ± SEM over sessions. Filled: direction rules; open: cue-based rules.

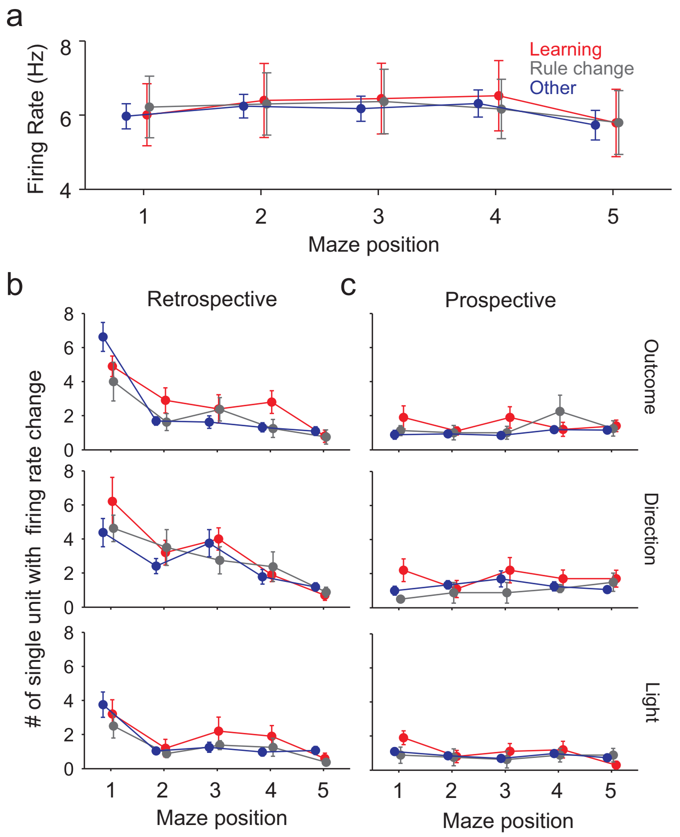

Supplementary Figure 10. Weak single neuron tuning to retrospective and prospective task elements.

(**a**) Firing rate of all neurons of the core population within a session type, at each maze position. Symbols give means ± SEM. There is no position or session-type selective effect on overall neuron activity, consistent with the findings of Fujisawa et al (2008, Nature Neurosci, 11, 823). (Maze positions 1: reward location; 3: choice point; 5: return to start arm).

(**b**) Number of tuned neurons for each maze position, for the preceding outcome (top), choice of direction (middle), and cue position (bottom). Only a few neurons in the core population showed tuning to any preceding task feature, with no differences between session types on any task element. Symbols give mean ± SEM over all neurons within a session type.

(**c**) As for panel **b**, for the prospective encoding of task elements.
